# Effectiveness of TES and rTMS for the Treatment of Insomnia: Meta-Analysis and Meta-Regression of Randomized Sham-Controlled Trials

**DOI:** 10.3389/fpsyt.2021.744475

**Published:** 2021-10-22

**Authors:** Haixia Ma, Jingxia Lin, Jiali He, Dilys Hoi Ting Lo, Hector W. H. Tsang

**Affiliations:** ^1^Department of Rehabilitation, The Hong Kong Polytechnic University, Kowloon, Hong Kong, SAR China; ^2^Mental Health Research Centre, The Hong Kong Polytechnic University, Kowloon, Hong Kong, SAR China

**Keywords:** insomnia, transcranial electric stimulation, repetitive transcranial magnetic stimulation, meta-analysis, meta-regression

## Abstract

**Objectives:** Transcranial electric stimulation (TES) and repetitive transcranial magnetic stimulation (rTMS) have experienced significant development in treating insomnia. This review aims to examine the effectiveness of randomized sham-controlled trials of TES and rTMS in improving insomnia and examine potential moderators associated with the effect of the treatment.

**Methods:** Nine electronic databases were searched for studies comparing the effects of TES/rTMS with sham group on insomnia from the inception of these databases to June 25, 2021, namely, Medline, Embase, PsycINFO, CINAHL, Cochrane Library, Web of Science, PubMed, ProQuest Dissertation and Thesis, and CNKI. Meta-analyses were conducted to examine the effect of TES and rTMS in treating insomnia. Univariate meta-regression was performed to explore potential treatment moderators that may influence the pooled results. Risk of bias was assessed by using the Cochrane Risk of Bias Tool.

**Results:** A total of 16 TES studies and 27 rTMS studies were included in this review. The pooled results indicated that there was no significant difference between the TES group and the sham group in improving objective measures of sleep. rTMS was superior to its sham group in improving sleep efficiency, total sleep time, sleep onset latency, wake up after sleep onset, and number of awakenings (all *p* < 0.05). Both TES and rTMS were superior to their sham counterparts in improving sleep quality as measured by the Pittsburgh Sleep Quality Index at post-intervention. The weighted mean difference for TES and rTMS were −1.17 (95% CI: −1.98, −0.36) and −4.08 (95% CI: −4.86, −3.30), respectively. Gender, total treatment sessions, number of pulses per session, and length of treatment per session were associated with rTMS efficacy. No significant relationship was observed between TES efficacy and the stimulation parameters.

**Conclusions:** It seems that TES and rTMS have a chance to play a decisive role in the therapy of insomnia. Possible dose-dependent and gender difference effects of rTMS are suggested.

## Introduction

As one of the most commonly reported sleep complaints, insomnia affects approximately 10–35% of the general population (1). According to the diagnosis criteria described in the fifth edition of the Diagnostic and Statistical Manual of Mental Disorders and the third edition of the International Classification of Sleep Disorders, insomnia disorder is a predominant complaint of dissatisfaction with sleep quantity or quality, associated with one (or more) of the following symptoms: difficulty initiating sleep, difficulty maintaining sleep, and early-morning awakening. The symptom is presented for at least three nights per week for at least 3 months. It is associated with distress or impairments in daytime function (2, 3). Insomnia could exist alone or be comorbid with other physical or psychiatric disorders, such as chronic pain, Parkinson's disease, cancer, anxiety, or depression (4–7). It decreases the psychological wellbeing and quality of life in people suffering from it and is frequently associated with mood disorders, driving accidents, and a greater prevalence of physical impairment (7, 8).

The cortical hyperarousal plays a central role in the etiology of insomnia. Many studies reported that people with insomnia have a higher level of physiological arousal (9, 10). Therefore, reducing the arousal level may facilitate sleep. Various therapeutic approaches have been used and investigated to improve the sleep of people with insomnia. Pharmacological treatments are proven to be effective and available but related to abuse, dependence, and adverse effects (11). Psychological and behavioral therapies, such as cognitive behavior therapy, targeting somatic and cognitive arousal, have demonstrated promising efficacy for relieving insomnia (12, 13) but remained underutilized due to highly demanding resources (14).

In recent years, non-invasive brain stimulation (NIBS) techniques have experienced significant development and gained increasing attention from researchers. Transcranial electric stimulation (TES) and repetitive transcranial magnetic stimulation (rTMS) are the two most popular types of NIBS. They share common characteristics of being relatively painless, safe, and well-tolerated with different mechanisms (15). TES is a neuromodulation approach that applies a low-intensity electrical current to the cerebral cortex of the brain. It includes cranial electrotherapy stimulation (CES), transcranial direct current stimulation (tDCS), transcranial alternative current stimulation (tACS), and transcranial random noise stimulation (tRNS). CES is a portable device that usually applies pulsed and low-level micro-current (<1 mA) stimulation to the brain *via* electrodes clipped onto the earlobes. It was approved by the US Food and Drug Administration for the treatment of insomnia, anxiety, and depression (16). tDCS modulates cortical activity by employing a constant, low-intensity current (0.5–2 mA) to the scalp over a pair of saline-sponge electrodes (17). The two electrodes are placed according to the international 10–20 electrode placement system (18). Generally speaking, anodal stimulation increases cortical excitability, while cathodal stimulation induces an opposite effect, i.e., reducing the cortical excitability (19). tACS and tRNS are relatively new TES techniques, which aim to increase the cortical excitability in a way similar to tDCS. However, instead of giving a steady and constant current between the two sites, tACS delivers a non-constant current to the brain so as to modulate the neural oscillations (20), and tRNS gives random frequencies between 0.1 and 640 Hz with a random noise distribution (21, 22). Currently, the mechanism of the effect of TES on insomnia is not well-established. It is hypothesized that TES could interfere with slow oscillation in the brain, which could increase the slow wave activity and enhance the low-frequency electroencephalogram (EEG) activity (i.e., a marker of arousal) (23–25). Due to its portable features and convenience to use, TES is suitable for self-administration at home (26, 27). However, TES is not free of limitations, and it has been criticized for poor spatial accuracy (28).

rTMS, on the other hand, is another appealing approach that combines both neurostimulation and neuromodulation techniques (29). It was developed in the 1980s and had shown therapeutic potential in improving insomnia (30, 31). Unlike TES, which stimulates the brain by delivering a weak current, rTMS utilizes electromagnetic induction. During rTMS stimulation, an electromagnetic coil is placed over the scalp. The coil could generate rapidly changing focal magnetic pulses that induce an electrical current to stimulate the neurons (29, 32, 33). rTMS has been classified into high (fast) frequency (>1 Hz) and low (slow) frequency (≤1 Hz) (34). rTMS at high frequency tends to have an excitatory effect, while rTMS at low frequency appears to have an inhibitory effect on the cortex (35). rTMS is regarded as a parameter-dependent technique. Its therapeutic effect could be influenced by the characteristics of the participants and a range of stimulation parameters, such as frequency, number of sessions, number of pulses/session, length of treatment/session, total number of pulses, and stimulation site (36, 37). Regarding the mechanism of rTMS on insomnia, it has been suggested that rTMS may reduce the state of hyperarousal and regulate brain plasticity by increasing the release of sleep-related hormones, such as brain-derived neurotrophic factor and gamma-aminobutyric acid (38, 39). rTMS is usually delivered in clinical settings. Compared to TES, rTMS has a better focality of stimulation and time resolution. Meanwhile, rTMS has significant limitations in terms of cost and poor portability. It also requires constant attention from the therapist during the treatment (40).

Although several reviews have been conducted to summarize the effectiveness of NIBS techniques for insomnia (15, 41–45), they were either narrative summaries on available evidence without meta-analysis (15, 41) or merely focused on one form of NIBS (42–45). Moreover, the moderators of the therapeutic effect of TES and rTMS on insomnia have not been extensively studied. Therefore, we aimed to review the therapeutic effects of TES and rTMS for the treatment of insomnia and investigate differences between them and the potential moderators associated with the treatment while restricting our review to randomized sham-controlled trials.

## Methods

### Search Strategy

Nine electronic databases were searched from the inception of these databases to June 25, 2021, including Medline, Embase, PsycINFO, CINAHL, Cochrane Library, Web of Science, PubMed, ProQuest Dissertation and Thesis, and CNKI. The retrieved abstracts and full-text articles were screened according to the PICOS framework.

The included studies should meet the following criteria: (1) Population: people with insomnia according to clinical diagnosis or had insomnia secondary to or comorbid with other physical or mental diseases or had a subjective complaint of insomnia without a clinical diagnosis; (2) Intervention: TES/rTMS techniques being employed as monotherapy or augmentation therapy for insomnia, such as TES/rTMS plus usual care or other types of intervention, were both eligible if the main aim of the study was to examine the effect of TES/rTMS and the sole difference between intervention and control was TES/rTMS. The search terms included transcranial electric stimulation or TES or cranial electrical stimulation OR CES OR cranial electric stimulat^*^ OR electrotherap^*^ OR fisher wallace stimulat^*^ OR alpha-stim OR Neuroelectric therapy OR Transcerebral electrotherapy OR Transcranial stimulation OR tDCS OR Brain Polarization OR Electric Stimulation OR Electric Polarization OR transcranial alternative current stimulation OR tACS OR transcranial random noise stimulation OR tRNS OR transcranial magnetic stimulation OR TMS OR non-invasive brain stimulation OR NIBS; (3) Comparison: studies compare TES/rTMS with a sham group; (4) Outcome: each study must have reported at least one of the following objective or subjective measurements of insomnia: sleep onset latency (SOL), total sleep time (TST), wake after sleep onset (WASO), sleep efficiency (SE), number of awakenings (NA), or subjective sleep quality—for example, polysomnography (PSG) is considered a “gold standard” for the diagnosis of sleep disorders. The Pittsburgh Sleep Quality Index (PSQI) and Insomnia Severity Index (ISI) are popular subjective instruments of sleep quality and severity of sleep disturbance; and (5) Study design: only randomized controlled trial (RCT) was included. The search was limited to articles in English and Chinese languages. Studies which failed to meet the abovementioned inclusion criteria were excluded.

Two authors (JL and DL) independently screened the title, abstract, and full text of the studies and determined the study eligibility. Any disagreement was resolved by consensus through a discussion or further consultation with a third author (HX) if needed.

### Data Extraction

Two authors independently extracted data from the included articles. The characteristics of the study were extracted and tabulated according to authors, year of publication, country, types of insomnia, diagnosis, age, percentage of males, sample size, attrition rate, treatment parameters, sham procedure, main instruments used for outcome measurements, and assessment time point. The treatment parameters included electrode/coil position, current intensity, stimulation frequency, magnetic field strength, (resting) motor threshold, number of pulses per session, duration of the treatment per session, and total number of sessions.

### Assessment of the Risk of Bias

The risk of bias of the included study was assessed using the Cochrane Risk of Bias Tool. The assessment was done across seven domains of bias: (1) random sequence generation, (2) allocation concealment, (3) blinding of participants and personnel, (4) blinding of care providers, (5) blinding of outcome assessment, (6) incomplete outcome data, and (7) selective reporting. Each study was ranked as having low, high, or unclear risk of bias for each of the potential sources of bias. Discrepancies were discussed until a consensus was reached.

### Statistical Analysis

The data was analyzed using the Review Manager (version 5.4). Changes in the continuous outcome were expressed as weighted mean difference (WMD) when the outcome was measured with the same scale. Otherwise, standard mean difference was used. Changes in dichotomous outcomes were expressed as relative risks (RR). The corresponding 95% confidence interval (CI) was calculated. To estimate the statistical heterogeneity of the intervention effects among studies, the *I*^2^ statistic was used, in which *I*^2^ < 25%, 25–50%, and >50% were considered low, moderate, and high heterogeneity, respectively. Fixed-effects model was performed to calculate the pooled mean difference if *I*^2^ < 50%. Otherwise, random-effects model was performed. When data was available, immediate, short-term, and long-term effects were also analyzed and compared. In this review, the immediate follow-up was defined as 0 to <1 week post-intervention, the short-term follow-up was defined as 1 to 4 weeks post-intervention, and the long-term follow-up was defined as >4 weeks post-intervention. To further explore the heterogeneity of the results, sensitivity analyses were limited to studies among participants with primary insomnia and having a lower or unclear risk of bias.

To explore potential treatment moderators that may influence the pooled results, univariate meta-regression of continuous moderators was performed using Comprehensive Meta-Analysis software (version 3.0). The analyses were restricted to studies with at least six effect sizes for a continuous variable and four effect sizes per group for a categorical variable (46). The following possible moderators were considered: mean age of the participants, percentage of males, stimulation intensity (milliampere), frequency (Hz), number of pulses per session, total number of sessions, number of weekly sessions, length of each session, and stimulation site. Multivariable meta-regression analyses were not conducted to avoid exceeding the power of the pooled studies (47). All *p*-values were set at 0.05 level (two-tailed).

In the presence of potential publication bias, funnel plots and Egger's regression test were applied using Comprehensive Meta-Analysis software (version 3.0). The funnel plots were analyzed when at least 10 studies were included in the meta-analysis.

## Results

**Figure 1:**
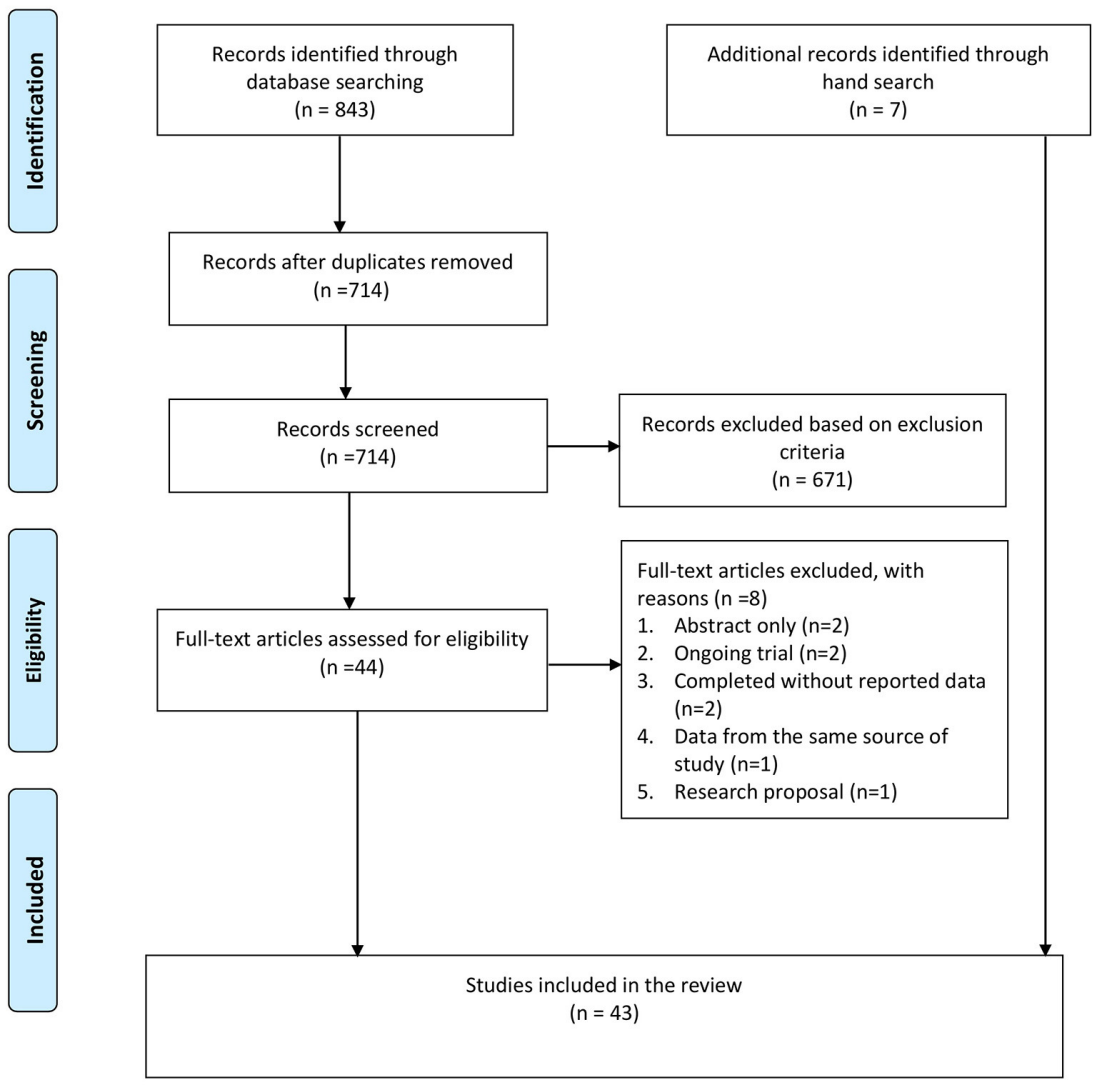
PRISMA flow chart. Adapted from Moher et al. (48).

A total of 843 citations were identified from the databases, and 129 duplicates were removed. After screening the title and abstract, 44 full-text articles were retrieved for further assessment. Of these, eight studies were excluded for the following reasons: abstract without full text (*n* = 2), ongoing trial without outcome data (*n* = 2), completed RCT without reporting data (*n* = 2), duplicated publication with data from the same source of study (*n* = 1), and research proposal (*n* =1). Besides these, seven additional studies were identified from the hand search of the reference lists. In total, 43 studies were included in this review, including 16 TES studies and 27 rTMS studies (see Figure 1).

Among the TES studies, eight of them used CES (49–56), seven applied tDCS (57–63), and one examined tACS (64). No study was identified to have examined the effectiveness of tRNS in insomnia. A summary of the characteristics of TES and rTMS is described in the following section.

### Characteristics of TES Studies

#### Participants

The groups of individuals treated by TES were heterogeneous, including people with cancer, fibromyalgia, post-polio, HIV, substance abuse, or women after menopause. The diagnosis of insomnia varied across studies. Of nine studies that reported a diagnosis of insomnia, three used DSM, ICSD, or International Statistical Classification of Diseases and Related Health Problems (ICD-10) criteria, and three studies used the cutoff scores of PSQI, ISI, and General Sleep Disturbance Scale (GSDS). One study employed the diagnosis of neurotic and personality disorders combined with insomnia, and one study adopted sleep parameters to determine insomnia. The sample size in each study ranged from 10 to 167, with a median number of 32. The mean age of all participants was 52.5 (SD = 7.36) years. There were considerably more female subjects than male subjects, with the male-to-female ratio being 1:3.2. The attrition rate ranged from 0.0 to 19.3%.

#### Electrode Position

Usually, most CES studies used ear-clip electrodes attached to the earlobes (*n* = 5). All tDCS studies applied one anode and one cathode, except that one study (57) used two anodes and one cathode. The anode was located according to the International 10–20 EEG system. Four studies applied the anode over the C3/C4 area. Three tDCS studies located the anode at the right or left dorsolateral prefrontal cortex (DLPFC) area, and one study placed the anode at the right inferior frontal cortex near F10. The cathode locations also varied. Three studies located the cathode at the contralateral supraorbital region, two studies chose the right/left DLPFC area, one study put it on the left shoulder, and another study placed it on the contralateral upper arm. The tACS study was composed of three electrodes; one was placed over the forehead, and two others were placed over the mastoid area (see Supplementary Table 1).

#### Stimulation Parameters

A low current of 0.1 mA was used in the majority of CES studies. The current intensity ranged from 1.5 to 2 mA in the tDCS studies and was 15 mA in the tACS study. The dosage and follow-up frame of the intervention varied widely. The duration of each session lasted from 5 to 90 min, with the majority of CES studies lasting for 60 min and of tDCS studies lasting for 20 min. The majority of TES was administered once daily for a duration of 5 days to 4 weeks. The majority of the studies only measured the outcome immediately after the end of the intervention. Five studies collected follow-up assessment at 1–4 weeks post-intervention, and one study investigated the effect of CES at 2 years of follow-up (see Supplementary Table 1).

#### Sham TES Procedure

All TES studies used a similar type of sham procedure, which involved no electrical current or gave a few seconds of electrical stimulation at the beginning/end of the intervention.

### Characteristics of rTMS Studies

#### Participants

As summarized in Supplementary Table 2, a total of 27 studies applied rTMS technique (65–91). The diagnosis of insomnia also differed. Of 25 studies that reported the diagnosis of insomnia, 14 studies used the DSM or ICD criteria, six studies used the Chinese Classification of Mental Disorders, three studies adopted the diagnosis and treatment of adult insomnia in China, and two studies employed the cutoff score of PSQI. Most rTMS studies include patients with primary insomnia (*n* = 18). The sample size in each study varied from 19 to 160, with a median number of 78. The mean age of all participants was 47.5 (SD = 10.26), and the male-to-female ratio was 1:1.3. The attrition rate ranged from 0.0 to 13.3%.

#### Stimulation Site

Regarding rTMS trials, the majority of them targeted the right DLPFC (*n* = 19). Other sites included the left prefrontal cortex (PFC) (*n* = 2), the right lateral and middle PFC (*n* = 1), the vertex (*n* = 1), the right posterior parietal cortex (P4 electrode site) (*n* = 1), the raphe nuclei (*n* = 1), the middle of the bilateral frontal/occipital/temple cortex (*n* = 1), and certain acupoints (*n* = 1). One rTMS study did not describe the stimulation site (see Supplementary Table 2).

#### rTMS Stimulation Parameters

The intensity of the rTMS studies also varied. The stimulation frequency in most studies ranged from 0.5 to 1 Hz, the stimulation intensity ranged from 80 to 130% motor threshold, and the number of pulses per session ranged from 1,100 to 2,400. The duration of each session lasted from 10 to 90 min, with the majority of interventions lasting for 20 min (*n* = 15). The majority of rTMS was administered on consecutive days or 5 days per week for a duration of 2 to 4 weeks. Most studies measured the outcome immediately after the intervention. Seven studies also collected data at 1–22 weeks of follow-up (see Supplementary Table 2).

#### Sham rTMS Procedure

The common sham methods applied in rTMS studies were using a 90°/180° tilted coil (*n* = 13) or an inactive coil with/without a sound effect (*n* = 11).

### Outcome Measurements of TES and rTMS Studies

Various measures have been applied in the included studies. Laboratory-based PSG, sleep diary, EEG, and actigraphy provided an objective evaluation of the sleep parameters. The most popular objective measurement was PSG-measured SOL (*n* =13), followed by PSG-measured WASO (*n* = 10), PSG-measured SE (*n* = 9), PSG-measured TST (*n* = 9), and PSG-measured NA (*n* = 6). Among the subjective measurements of sleep, the PSQI was the most frequently used measurement (*n* = 33), followed by the GSDS (*n* = 3), ISI (*n* = 2), and Krakow Sleep Score (*n* = 1) (see Supplementary Tables 1, 2).

### Quality Assessment

Overall, the risk of bias of the included studies was considered mediocre. The majority of the studies failed to report a detailed methodology. Moreover, 23 of them did not report an adequate method of random sequence generation. Only three studies described the allocation concealment. All studies were rated as having a low risk of bias in blinding the participants because of the sham procedure. However, it was difficult to blind the practitioner of the assigned intervention in most of the RCTs. Only one study blinded the practitioner *via* adopting pre-set sham devices provided by a device manufacturer. A total of 14 studies reported blinding of outcome assessment, while the rest of the 29 studies did not. Regarding the outcome data, 39 studies were considered as having a low risk of attrition bias (dropout rate <10%, used intention-to-treat analysis). In comparison, a high risk of attrition bias was reported in the remaining four studies (dropout rate >10%). All studies, except one, reported complete outcome data (see Supplementary Figures 1, 2).

### Synthesis of Results

#### TES Studies

##### 1) Objective Measures of Sleep Parameters

Supplementary Table 3 presents the results of a meta-analysis on PSG and EEG measures of sleep parameters in TES studies. Two RCTs evaluated the effectiveness of TES on SE and total TST, and three RCTs reported the results of TES on SOL. The findings from the random-effects model indicated that there was no significant difference between the TES group and the sham group in improving SE (WMD: −4.86, 95% CI: 17.29, 7.57, *p* = 0.0003), TST (WMD: −7.75, 95% CI: −42.25, 26.74, *p* = 0.66), or SOL (WMD: 1.24, 95% CI: −10.05, 12.52, *p* = 0.83). Between studies, substantial heterogeneity among these sleep parameters existed, which ranged from 79 to 95%.

##### 2) Subjective Measurement of Sleep Quality—PSQI

**Figure 2:**
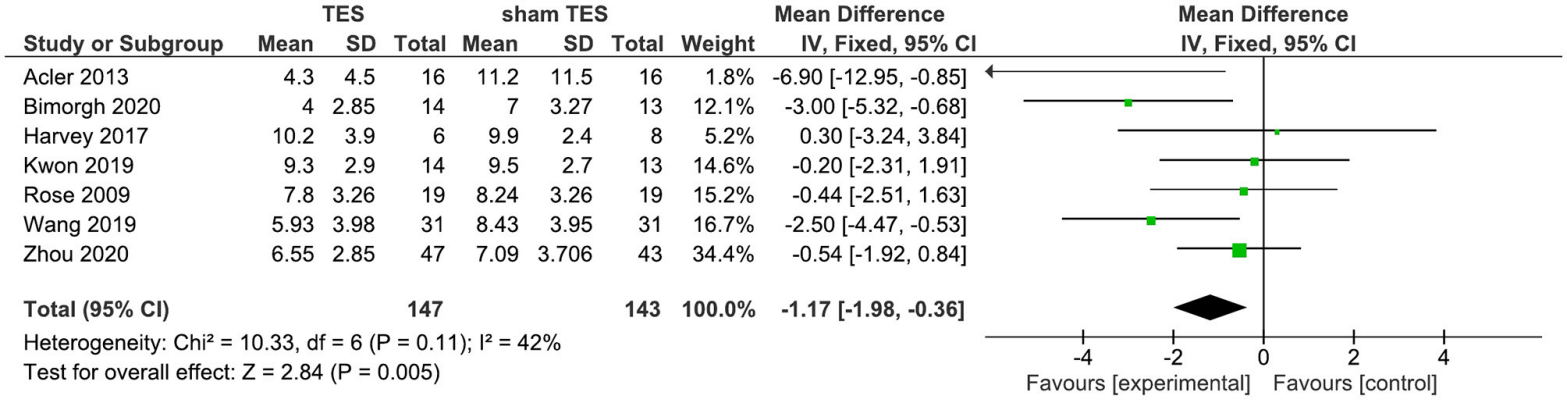
TES vs. sham TES on PSQI at the post intervention.

A total of seven studies reported that the results of TES contrast with those of sham TES in terms of changes of the PSQI total score immediately after the intervention. Findings from the fixed-effects model showed that active TES were superior to their sham counterparts in improving the PSQI total score. The WMD for TES was −1.17 (95% CI: −1.98, −0.36) (Figure 2). Nevertheless, moderate heterogeneity existed among TES studies (*I*^2^ = 42%).

Supplementary Table 4 displays the results of univariate analyses of moderators for effects of TES on sleep quality as measured by PSQI. The results of the meta-regression of TES studies revealed that age, percentage of males, current intensity, total number of treatment sessions, number of weekly sessions, and duration of each session were not significant moderators for the effects of TES on sleep quality as measured by the PSQI.

#### rTMS Studies

##### 1) Objective Measures of Sleep Parameters

The results of the meta-analyses on PSG and the actigraphy measures of SE, TST, SOL, WASO, and NA in rTMS studies are shown in Supplementary Table 5. Eight studies reported on the effectiveness of rTMS on SE and TST. The pooled results indicated that rTMS was superior to the sham group in improving SE (random-effects model: WMD −7.91; 95% CI −3.70, 12.12; *p* < 0.00001) and TST (random-effects model: WMD −37.25, 95% CI −21.51, 52.98). A total of 12 studies reported the effect of rTMS on SOL, and the WMD for rTMS was −9.78 (95% CI: −13.25, −6.31). Eleven studies examined the effect of rTMS on WASO. The findings from the random-effects model indicated that rTMS was superior to the sham counterpart in improving WASO (random-effects model: WMD: −27.86; 95% CI: −38.70, −17.02; *p* < 0.00001). However, the pooled data on SE, TST, SOL, and WASO had substantial heterogeneity, and the *I*^2^ ranged from 80 to 96%. In addition, seven studies evaluated the effect of rTMS on NA. According to the fixed-effects model, rTMS significantly reduced the NA (WMD: −1.06; 95% CI:−1.53, 0.59; *p* < 0.00001). Mild heterogeneity between studies was found (*I*^2^ = 22%).

##### 2) Subjective Measurements of Sleep Quality—PSQI

**Figure 3:**
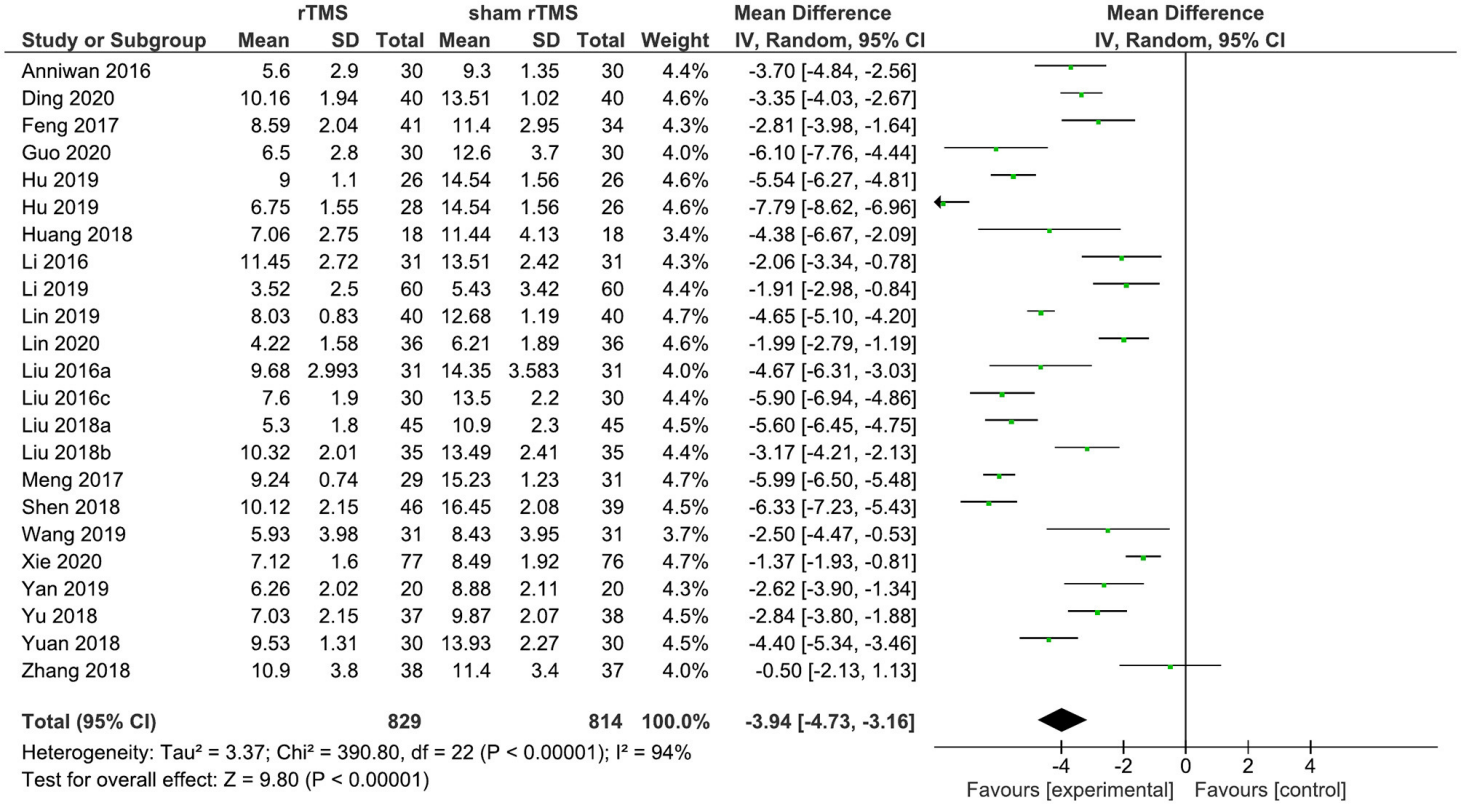
rTMS vs. sham rTMS on PSQI at the post intervention.

A total of 22 rTMS studies provided data on the PSQI total score after the completion of the intervention. The meta-analysis showed the evidence of a positive effect of rTMS on sleep quality compared to the sham group (WMD: −4.08; 95% CI: −4.86, −3.30, *p* < 0.00001). However, pronounced heterogeneity was also recorded between studies (*I*^2^ = 94%; Figure 3).

**Figure 4:**
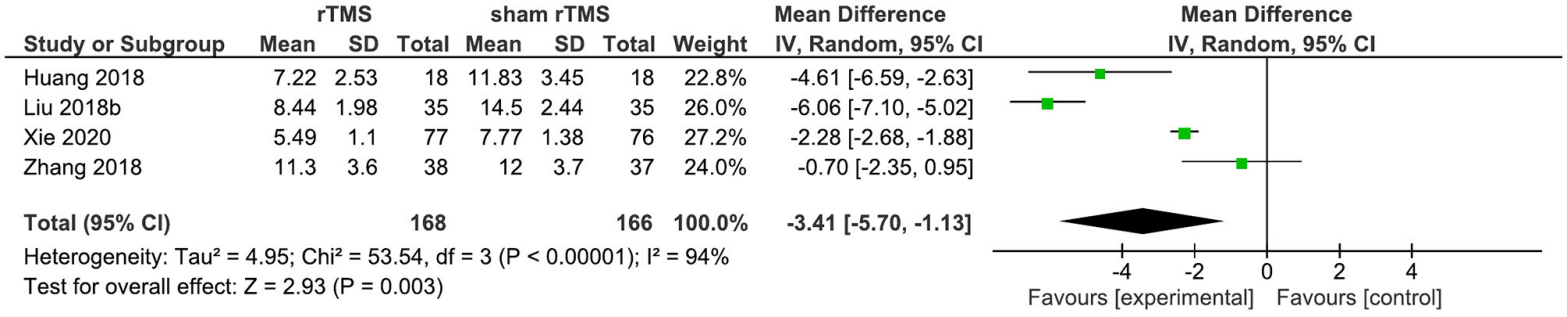
rTMS vs. sham rTMS on PSQI at 1–4 weeks follow up.

Four studies provided data on the PSQI total score in the short term (1–4 weeks post-intervention). rTMS, as compared to sham rTMS, resulted in a statistically significant improvement in the PSQI total score (WMD: −3.41; 95% CI: −5.70, −1.13; *p* = 0.003). Significant heterogeneity existed (*I*^2^ = 94%; Figure 4).

Supplementary Table 6 displays the results of the univariate analyses of moderators for the effects of rTMS on insomnia. The results of the meta-regression of rTMS studies showed that a greater number of total treatment sessions was associated with a greater improvement in SE and PSQI score (both *p* < 0.05). The results also indicated a significant inverse relationship between the length of rTMS treatment per session and the improvement in the TST and PSQI scores (both *p* < 0.05). Male gender and a greater number of pulses per session were associated with a significant improvement in the PSQI total score at post-intervention (all *p* < 0.05), whereas age and stimulation site were insignificant.

### Sensitivity Analysis

Regarding the high level of heterogeneity in the studies, especially in TES studies, we restricted the meta-analysis to participants with primary insomnia and the results of the sensitivity analysis by removing five TES studies that confirmed the findings of the entire dataset of seven TES studies (see Supplementary Figure 3). The results of the sensitivity analysis by removing four rTMS studies also confirmed the findings of the entire dataset of 22 rTMS studies (see Supplementary Figure 4). Furthermore, the sensitivity analysis, by excluding one TES study and two rTMS studies with a high risk of bias, did not change the overall estimate of the effects of TES and rTMS (see Supplementary Figures 5, 6).

### Publication Bias

Egger's test was used to assess the publication bias for the effect of rTMS on the objective and subjective measures of insomnia. Egger's test indicated that there was no evidence of significant publication bias in rTMS studies that reported WASO (intercept = −12.90; two-tailed 95% CI, −32.89, 7.09; *p* = 0.18) (Supplementary Figure 7), while potential publication bias was found in rTMS studies that reported SOL (intercept = −10.02; two-tailed 95% CI, −19.69, −0.34; *p* = 0.04) (Supplementary Figure 8) and PSQI score (intercept = −11.77; two-tailed 95% CI, −14.99, −8.56; *p* = 0.000) (Supplementary Figure 9).

### Adverse Events

Mild and temporary adverse events were reported in tDCS and rTMS studies. The most frequently observed adverse events in tDCS studies were dizziness, discomfort, or itching at the stimulation site. The frequently reported adverse events in rTMS studies were headache, dizziness, pain at the stimulation site, discomfort, itchiness, muscle spasm, and constipation (see Supplementary Table 7).

The meta-analyses of the safety outcomes are summarized in Supplementary Table 8. The occurrence of any adverse events, dizziness, or headache did not differ significantly between the NIBS group and the sham NIBS group and in their subgroup analysis. However, a marginally significant association was found between NIBS and the complaints of discomfort by the participants (RR = 5.00; 95% CI: 0.89, 27.97; *p* = 0.07, *I*^2^ = 0%). Furthermore, pain was a common side effect of rTMS and was reported in six studies. The participants in the rTMS group were significantly more likely to experience more pain at the stimulation site than those in the sham rTMS group (RR = 2.58; 95% CI: 1.14, 5.84; *p* = 0.02, *I*^2^ = 0%).

## Discussion

This review extends and improves previous reviews on the effectiveness of NIBS on insomnia. It examined the effects of TES and rTMS using a meta-analytical approach in treating insomnia and examining the potential moderators associated with the treatment. Overall, both techniques could be, respectively, considered as an effective and safe approach for insomnia, while the data suggested a greater effect size with rTMS than TES in improving SE, SOL, TST, and PSQI total score. In the following section, the possible explanations of differences in the treatment effectiveness between TES and rTMS, the dose-dependent effect of rTMS, and the gender difference in the effect of rTMS are discussed.

Findings from our review support the use of TES for insomnia. TES was superior compared to their sham counterparts in improving PSQI total score. However, it failed to demonstrate superiority in objective measures of sleep parameters. Compared to rTMS, TES also showed less strong evidence in improving sleep-related outcomes. The differences in effectiveness may be explained by a number of factors. Firstly, given the differences in the characteristics of the participants and the intervention, clinical heterogeneity should be considered. Secondly, the analysis was based on a relatively small number of TES studies. More TES studies should be conducted to confirm the superiority of either approach. Thirdly, the underlying mechanism of the two techniques could be another possible explanation for the difference in the therapeutic outcomes. For TES, it was assumed that only some fractions of the current could pass through the scalp. For rTMS, the magnetic field generated by stimulating the coil could pass through the scalp directly and reach the deep cortex cortical without energy loss (37, 92). The mechanism underlying the therapeutic effects of TES and rTMS need further exploration, while the findings from this review are still encouraging since TES devices have many advantages and have the potential to be easily promoted in the community—for example, most TES devices are portable and wearable, and they could even be self-administered at home by people with sleep problems. With the portable character, their effect on facilitating sleep could be enlarged.

The results of this review are in line with the prior meta-analysis that rTMS is effective in improving PSQI (43). Furthermore, the results from this review extend our knowledge of rTMS in improving objective sleep parameters, including SE, SOL, TST, WASO, and NA. Regarding the stimulation parameters, the included studies ranged from 10 to 30 sessions, with each session consisting of 1,100 to 2,000 pulses for 10 to 30 min. The finding from this review suggests a potential dose-dependent effect of rTMS in treating insomnia. A greater number of treatment sessions is associated with better SE and sleep quality as measured by PSQI, and a greater number of pulses per session is associated with an improved PSQI. These findings are consistent with many previous studies among people with other mental disorders (93–97). Meanwhile, the inverse associations between the length of treatment per session and TST and PSQI are also noteworthy. There is evidence that the rTMS technique could induce a cumulative effect on cortical excitability that outlasts the stimulation period (98). However, prolonged rTMS stimulation could have a reversed after-effect (99, 100). The abovementioned findings raise several interesting questions about designing an optimal rTMS treatment protocol for people with insomnia: What is the optimal number of pulses per session/duration of stimulation per session/total number of sessions? Does the cumulative number of pulses show the same relationship with the therapeutic outcome as the cumulative number of sessions? How long will the cumulative effect last?

Previous studies on gender differences have reported that gender, age, and menopausal status could predict rTMS response (101, 102). This review also showed that male subjects had a higher response to rTMS treatment in insomnia as measured by the PSQI. We speculate that such differences may be attributed to age and the associated level of sex hormones. As significant confounding factors for the association between gender and rTMS response, older age and the menopausal status of females could predict worse rTMS response (101, 102). In general, the average age of menopause is approximately 52.5 years (103), and the average age of the participants in our pooled analysis was 50.59 (SD = 11.05) years. It is thus plausible that a significant number of women were in the stage of perimenopause or menopause, resulting in a decreased rTMS response in females. However, due to the lack of a detailed description of the clinical characteristics of the participants, especially the age of male and female subjects, the gender ratio in the active rTMS group compared to the sham group, and menopausal status, future studies are recommended to explore the influence of confounding factors, such as age, gender, and sex hormones level, on rTMS response among people with insomnia.

### Strengths and Limitations

One of the strengths of this review is that it examined two forms of NIBS technique with more RCTs and more participants. Furthermore, it described the trends in outcomes across the immediate post-intervention and short-term follow-up and considered the moderators of effects of TES and rTMS.

There are also some limitations in this review. Firstly, due to the poor reporting of random sequence generation and allocation concealment in most of the included studies, it was difficult to evaluate the methodology quality. Secondly, substantial heterogeneity existed, which may partially be explained by the differences in the characteristics of the participants, diagnosis of insomnia, and stimulation parameters. This review also included participants with insomnia comorbid with other chronic conditions without control by the use of medication. The results should be interpreted with caution. Thirdly, due to the lack of studies evaluating the long-term effect of TES and rTMS in treating insomnia, only the short-term effect could be examined. The small number of studies may also limit the generalization of the findings. To further elucidate whether the effects could be sustained over time, future studies are suggested to adopt a longer follow-up period. The ideal follow-up period may be 3 months as insomnia is characterized by the sleep difficulty symptom that lasts for at least 3 months.

## Conclusion

Overall, TES and rTMS are promising approaches in improving the symptoms of insomnia. rTMS was better studied and showed a larger effect size than TES in both the objective and subjective measures of sleep, with therapeutic effect maintained at 1–4 weeks of follow-up. Individual characteristics and stimulation parameters, such as gender, number of pulses per session, total number of treatment sessions, and length of treatment per session, were associated with the effect of rTMS and should be considered when developing optimal treatment protocols. This review highlighted the paucity of research on TES study. Future research with a longer follow-up period is also recommended.

## Data Availability Statement

The original contributions presented in the study are included in the article/Supplementary Material, further inquiries can be directed to the corresponding author/s.

## Author Contributions

HT initiated the idea and conceptualized the framework for the article. JH and DL performed the literature search and data extraction. HM conducted the data synthesis and wrote the first draft of the manuscript. JL and HT commented on previous versions of the manuscript. All authors have read and approved the final manuscript.

## Conflict of Interest

The authors declare that the research was conducted in the absence of any commercial or financial relationships that could be construed as a potential conflict of interest.

## Publisher's Note

All claims expressed in this article are solely those of the authors and do not necessarily represent those of their affiliated organizations, or those of the publisher, the editors and the reviewers. Any product that may be evaluated in this article, or claim that may be made by its manufacturer, is not guaranteed or endorsed by the publisher.
