# Supplementary material for: Effectiveness of TES and rTMS for the Treatment of Insomnia: Meta-Analysis and Meta-Regression of Randomized Sham-Controlled Trials
Source: Front Psychiatry. 2021 Oct 22;12:744475. doi: 10.3389/fpsyt.2021.744475 (PMC8569107; doi:10.3389/fpsyt.2021.744475)
Supplement: Supplementary file 1 [file Data_Sheet_1.docx]

**Supplementary data**

**Literature search strategies: Search Date: 25/2/2021**

| **Databases** |  | **Search terms** | **Items found** |
| --- | --- | --- | --- |
| Medline |  | TX insomnia or dyssomnia or sleep disorders or sleep disturbance or sleeplessness or sleep maintenance or somnipathy | 76,250 |
|  |  | TX transcranial electric stimulation or TES or cranial electrical stimulation OR CES OR cranial electric stimulat* OR electrotherap* OR fisher wallacestimulat* OR alpha-stim OR Neuroelectric therapy OR Transcerebral electrotherapy OR Transcranial stimulation OR tDCS OR Brain Polarization OR Electric Stimulation OR Electric Polarization OR transcranial alternative current stimulation OR tACS OR transcranial random noise stimulation OR tRNSOR transcranial magnetic stimulation OR TMS OR non-invasive brain stimulation OR NIBS | 177,418 |
|  |  | TX (rct or randomized control trial or randomized controlled trial) OR (placebo or sham or control) | 4,851,014 |
|  |  | *1 AND 2 AND 3* (207) |  |
| Embase |  | insomnia:ti,ab,kw OR dyssomnia:ti,ab,kw OR 'sleep disorders':ti,ab,kw OR 'sleep disturbance':ti,ab,kw OR sleeplessness:ti,ab,kw OR 'sleep maintenance':ti,ab,kw OR somnipathy:ti,ab,kw | 66,110 |
|  |  | **'**transcranial electric stimulation**'**:ti,ab,kwor **'**TES**'**:ti,ab,kwor **'**cranial electrical stimulation**'**:ti,ab,kwOR **'**CES**'**:ti,ab,kwOR **'**cranial electric stimulat***'**:ti,ab,kw OR **'**electrotherap***'**:ti,ab,kw OR **'**fisher wallacestimulat***'**:ti,ab,kwOR **'**alpha-stim**'**:ti,ab,kw OR **'**Neuroelectric therapy**'**:ti,ab,kwOR **'**Transcerebral electrotherapy**'**:ti,ab,kwOR **'**Transcranial stimulation**'**:ti,ab,kw OR **'**tDCS**'**:ti,ab,kwOR **'**Brain Polarization**'**:ti,ab,kw OR **'**Electric Stimulation**'**:ti,ab,kw OR **'**Electric Polarization**'**:ti,ab,kwOR**'**transcranial alternative current stimulation**'**:ti,ab,kwOR **'**tACS**'**:ti,ab,kw OR **'**transcranial random noise stimulation**'**:ti,ab,kw OR **'**tRNS**'**:ti,ab,kwOR **'**transcranial magnetic stimulation**'**:ti,ab,kwOR **'**TMS**'** OR **'**non-invasive brain stimulation**'**:ti,ab,kwOR **'**NIBS**'**:ti,ab,kw | 55,137 |
|  |  | rct OR 'randomized control trial' OR (randomized AND ('control'/exp OR control) AND ('trial'/exp OR trial)) OR 'randomized controlled trial'/exp OR 'randomized controlled trial' OR (randomized AND controlled AND ('trial'/exp OR trial)) | 913,433 |
|  |  | 'placebo'/exp OR placebo OR sham OR 'control'/exp OR control | 4,955,769 |
|  |  | *1 AND 2 AND 3 AND 4* | 55 |
| PsycINFO |  | [ab(insomnia or dyssomnia or sleep disorders or sleep disturbance or sleeplessness or sleep maintenance or somnipathy)](https://search.proquest.com/recentsearches.recentsearchtabview.recentsearchesgridview.scrolledrecentsearchlist.checkdbssearchlink:rerunsearch/2B6EA4A2589C4F36PQ/None?site=psycinfo&t:ac=RecentSearches) | 32,917 |
|  |  | ab(transcranial electric stimulation or TES) or ab(cranial electrical stimulation OR CES OR cranial electric stimulat* OR electrotherap* OR fisher wallacestimulat* OR alpha-stim OR Neuroelectric therapy) OR ab(transcerebral electrotherapy) ab(transcranial magnetic stimulation or tms ) OR ab(tdcs or transcranial direct current stimulation) or ab(Brain Polarization OR Electric Stimulation OR Electric Polarization) or ab(transcranial alternative current stimulation OR tACS) OR ab(transcranial random noise stimulation OR tRNSOR transcranial magnetic stimulation or TMS) OR ab(non-invasive brain stimulation or NIBS) | 26,849 |
|  |  | ab(rct or randomized control trial or randomized controlled trial) | 42,181 |
|  |  | [ab(placebo or sham or control)](https://search.proquest.com/recentsearches.recentsearchtabview.recentsearchesgridview.scrolledrecentsearchlist.checkdbssearchlink:rerunsearch/867BF51BB2724EAAPQ/None?site=psycinfo&t:ac=RecentSearches) | 570,274 |
|  |  | *1 AND 2 AND 3 AND 4* | 21 |
| CINAHL |  | 1. AB (insomnia or dyssomnia or sleep disorders or sleep disturbance or sleeplessness or sleep maintenance or somnipathy) | 16,811 |
|  |  | AB(transcranial electric stimulation or TES) or ab(cranial electrical stimulation OR CES OR cranial electric stimulat* OR electrotherap* OR fisher wallacestimulat* OR alpha-stim OR Neuroelectric therapy) OR ab(transcerebral electrotherapy) ab(transcranial magnetic stimulation or tms ) OR ab(tdcs or transcranial direct current stimulation) or ab(Brain Polarization OR Electric Stimulation OR Electric Polarization) or ab(transcranial alternative current stimulation OR tACS) OR ab(transcranial random noise stimulation OR tRNSOR transcranial magnetic stimulation or TMS) OR ab(non-invasive brain stimulation or NIBS) | 11,986 |
|  |  | TX (rct or randomized control trial or randomized controlled trial) | 77,496 |
|  |  | TX (placebo or sham or control) | 504,609 |
|  |  | *1 AND 2 AND 3 AND 4* | 13 |
| Cochrane Library |  | ti,ab,kw(insomnia or dyssomnia or sleep disorders or sleep disturbance or sleeplessness or sleep maintenance or somnipathy or sleep hygiene) | 24,844 |
|  |  | ti,ab,kw (transcranial electric stimulation or TES or cranial electrical stimulation OR CES OR cranial electric stimulat* OR electrotherap* OR fisher wallacestimulat* OR alpha-stim OR Neuroelectric therapy OR Transcerebral electrotherapy OR Transcranial stimulation OR tDCS OR Brain Polarization OR Electric Stimulation OR Electric Polarization OR transcranial alternative current stimulation OR tACS OR transcranial random noise stimulation OR tRNSOR transcranial magnetic stimulation OR TMS OR non-invasive brain stimulation OR NIBS) | 21,627 |
|  |  | ti,ab,kw (rct or randomized control trial or randomized controlled trial) | 619,645 |
|  |  | ti,ab,kw (placebo or sham or control) | 1,040,527 |
|  |  | *1 AND 2 AND 3 AND 4* | 187 |
| Web of Science |  | TI=(insomnia or dyssomnia or sleep disorders or sleep disturbance or sleeplessness or sleep maintenance or somnipathy or sleep hygiene) | 30,777 |
|  |  | **TOPIC:** (transcranial electric stimulation or TES) OR **TOPIC:** (cranial electrical stimulation OR CES OR cranial electric stimulat* OR electrotherap* OR fisher wallacestimulat* OR alpha-stim OR Neuroelectrictherap) *OR* **TOPIC:** (tdcs or transcranial direct current stimulation) *OR* **TOPIC:** (transcranial magnetic stimulation or tms or rtms) *OR* **TOPIC:** (Transcranial stimulation or Electric Stimulation or Transcerebral electrotherapy) OR **TOPIC:** (Brain Polarization OR Electric Stimulation OR Electric Polarization) OR **TOPIC:** (transcranial alternative current stimulation OR Tacs) OR **TOPIC:** (transcranial random noise stimulation OR tRNS) OR **TOPIC:** (non-invasive brain stimulation OR NIBS) | 65,461 |
|  |  | **TOPIC:** (rct or randomized control trial or randomized controlled trial) | 423,040 |
|  |  | placebo or sham or control | 6,387,580 |
|  |  | 1 AND 2 AND 3 AND 4 | 18 |
| PubMed |  | "insomnia"[Title/Abstract] OR "dyssomnia"[Title/Abstract] OR "sleep disorders"[Title/Abstract] OR "sleep disturbance"[Title/Abstract] OR "sleeplessness"[Title/Abstract] OR "sleep maintenance"[Title/Abstract] OR "somnipathy"[Title/Abstract] | 43,090 |
|  |  | (("transcranial"[All Fields] OR "transcranially"[All Fields]) AND ("electric stimulation"[MeSH Terms] OR ("electric"[All Fields] AND "stimulation"[All Fields]) OR "electric stimulation"[All Fields])) OR ("tes"[Supplementary Concept] OR "tes"[All Fields] OR "tes"[All Fields]) OR (("cranially"[All Fields] OR "skull"[MeSH Terms] OR "skull"[All Fields] OR "cranial"[All Fields]) AND ("electric stimulation"[MeSH Terms] OR ("electric"[All Fields] AND "stimulation"[All Fields]) OR "electric stimulation"[All Fields] OR ("electrical"[All Fields] AND "stimulation"[All Fields]) OR "electrical stimulation"[All Fields])) OR "CES"[All Fields] OR (("cranially"[All Fields] OR "skull"[MeSH Terms] OR "skull"[All Fields] OR "cranial"[All Fields]) AND ("electricity"[MeSH Terms] OR "electricity"[All Fields] OR "electric"[All Fields] OR "electrical"[All Fields] OR "electrically"[All Fields] OR "electrics"[All Fields]) AND "stimulat*"[All Fields]) OR "electrotherap*"[All Fields] OR (("fisher"[All Fields] OR "fisher s"[All Fields] OR "fishers"[All Fields]) AND ("wallace"[All Fields] OR "wallace s"[All Fields]) AND "stimulat*"[All Fields]) OR "alpha-stim"[All Fields] OR (("neuroelectric"[All Fields] OR "neuroelectrical"[All Fields]) AND ("therapeutics"[MeSH Terms] OR "therapeutics"[All Fields] OR "therapies"[All Fields] OR "therapy"[MeSH Subheading] OR "therapy"[All Fields] OR "therapy s"[All Fields] OR "therapys"[All Fields])) OR ("Transcerebral"[All Fields] AND ("electric stimulation therapy"[MeSH Terms] OR ("electric"[All Fields] AND "stimulation"[All Fields] AND "therapy"[All Fields]) OR "electric stimulation therapy"[All Fields] OR "electrotherapies"[All Fields] OR "electrotherapy"[All Fields])) OR (("transcranial"[All Fields] OR "transcranially"[All Fields]) AND ("stimulate"[All Fields] OR "stimulated"[All Fields] OR "stimulates"[All Fields] OR "stimulating"[All Fields] OR "stimulation"[All Fields] OR "stimulations"[All Fields] OR "stimulative"[All Fields] OR "stimulator"[All Fields] OR "stimulator s"[All Fields] OR "stimulators"[All Fields])) OR ("transcranial direct current stimulation"[MeSH Terms] OR ("transcranial"[All Fields] AND "direct"[All Fields] AND "current"[All Fields] AND "stimulation"[All Fields]) OR "transcranial direct current stimulation"[All Fields] OR "tdcs"[All Fields]) OR (("brain"[MeSH Terms] OR "brain"[All Fields] OR "brains"[All Fields] OR "brain s"[All Fields]) AND ("polar"[All Fields] OR "polarisabilities"[All Fields] OR "polarisability"[All Fields] OR "polarisable"[All Fields] OR "polarisation"[All Fields] OR "polarisations"[All Fields] OR "polarise"[All Fields] OR "polarised"[All Fields] OR "polarising"[All Fields] OR "polarities"[All Fields] OR "polarity"[All Fields] OR "polarization"[All Fields] OR "polarizations"[All Fields] OR "polarize"[All Fields] OR "polarized"[All Fields] OR "polarizer"[All Fields] OR "polarizers"[All Fields] OR "polarizes"[All Fields] OR "polarizing"[All Fields] OR "polars"[All Fields])) OR ("electric stimulation"[MeSH Terms] OR ("electric"[All Fields] AND "stimulation"[All Fields]) OR "electric stimulation"[All Fields]) OR (("electricity"[MeSH Terms] OR "electricity"[All Fields] OR "electric"[All Fields] OR "electrical"[All Fields] OR "electrically"[All Fields] OR "electrics"[All Fields]) AND ("polar"[All Fields] OR "polarisabilities"[All Fields] OR "polarisability"[All Fields] OR "polarisable"[All Fields] OR "polarisation"[All Fields] OR "polarisations"[All Fields] OR "polarise"[All Fields] OR "polarised"[All Fields] OR "polarising"[All Fields] OR "polarities"[All Fields] OR "polarity"[All Fields] OR "polarization"[All Fields] OR "polarizations"[All Fields] OR "polarize"[All Fields] OR "polarized"[All Fields] OR "polarizer"[All Fields] OR "polarizers"[All Fields] OR "polarizes"[All Fields] OR "polarizing"[All Fields] OR "polars"[All Fields])) OR (("transcranial"[All Fields] OR "transcranially"[All Fields]) AND ("alternance"[All Fields] OR "alternances"[All Fields] OR "alternant"[All Fields] OR "alternants"[All Fields] OR "alternate"[All Fields] OR "alternated"[All Fields] OR "alternately"[All Fields] OR "alternates"[All Fields] OR "alternating"[All Fields] OR "alternation"[All Fields] OR "alternations"[All Fields] OR "alternative"[All Fields] OR "alternatively"[All Fields] OR "alternatives"[All Fields]) AND ("current"[All Fields] OR "current s"[All Fields] OR "currently"[All Fields] OR "currents"[All Fields]) AND ("stimulate"[All Fields] OR "stimulated"[All Fields] OR "stimulates"[All Fields] OR "stimulating"[All Fields] OR "stimulation"[All Fields] OR "stimulations"[All Fields] OR "stimulative"[All Fields] OR "stimulator"[All Fields] OR "stimulator s"[All Fields] OR "stimulators"[All Fields])) OR "tACS"[All Fields] OR ("transcranial direct current stimulation"[MeSH Terms] OR ("transcranial"[All Fields] AND "direct"[All Fields] AND "current"[All Fields] AND "stimulation"[All Fields]) OR "transcranial direct current stimulation"[All Fields] OR ("transcranial"[All Fields] AND "random"[All Fields] AND "noise"[All Fields] AND "stimulation"[All Fields]) OR "transcranial random noise stimulation"[All Fields]) OR "tRNS"[All Fields] OR ("transcranial magnetic stimulation"[MeSH Terms] OR ("transcranial"[All Fields] AND "magnetic"[All Fields] AND "stimulation"[All Fields]) OR "transcranial magnetic stimulation"[All Fields]) OR ("symp theory model simul"[Journal] OR "tms"[All Fields]) OR ("non-invasive"[All Fields] AND ("brain stimul"[Journal] OR ("brain"[All Fields] AND "stimulation"[All Fields]) OR "brain stimulation"[All Fields])) OR "NIBS"[All Fields] | 237,512 |
|  |  | "rct"[All Fields] OR (("random allocation"[MeSH Terms] OR ("random"[All Fields] AND "allocation"[All Fields]) OR "random allocation"[All Fields] OR "random"[All Fields] OR "randomization"[All Fields] OR "randomized"[All Fields] OR "randomisation"[All Fields] OR "randomisations"[All Fields] OR "randomise"[All Fields] OR "randomised"[All Fields] OR "randomising"[All Fields] OR "randomizations"[All Fields] OR "randomize"[All Fields] OR "randomizes"[All Fields] OR "randomizing"[All Fields] OR "randomness"[All Fields] OR "randoms"[All Fields]) AND ("controling"[All Fields] OR "controllability"[All Fields] OR "controllable"[All Fields] OR "controllably"[All Fields] OR "controller"[All Fields] OR "controller s"[All Fields] OR "controllers"[All Fields] OR "controlling"[All Fields] OR "controls"[All Fields] OR "prevention and control"[MeSH Subheading] OR ("prevention"[All Fields] AND "control"[All Fields]) OR "prevention and control"[All Fields] OR "control"[All Fields] OR "control groups"[MeSH Terms] OR ("control"[All Fields] AND "groups"[All Fields]) OR "control groups"[All Fields]) AND ("clinical trials as topic"[MeSH Terms] OR ("clinical"[All Fields] AND "trials"[All Fields] AND "topic"[All Fields]) OR "clinical trials as topic"[All Fields] OR "trial"[All Fields] OR "trial s"[All Fields] OR "trialed"[All Fields] OR "trialing"[All Fields] OR "trials"[All Fields])) OR ("randomized controlled trial"[Publication Type] OR "randomized controlled trials as topic"[MeSH Terms] OR "randomized controlled trial"[All Fields] OR "randomised controlled trial"[All Fields]) OR ("placeboes"[All Fields] OR "placebos"[MeSH Terms] OR "placebos"[All Fields] OR "placebo"[All Fields]) OR ("salicylhydroxamic acid"[Supplementary Concept] OR "salicylhydroxamic acid"[All Fields] OR "sham"[All Fields]) OR ("controling"[All Fields] OR "controllability"[All Fields] OR "controllable"[All Fields] OR "controllably"[All Fields] OR "controller"[All Fields] OR "controller s"[All Fields] OR "controllers"[All Fields] OR "controlling"[All Fields] OR "controls"[All Fields] OR "prevention and control"[MeSH Subheading] OR ("prevention"[All Fields] AND "control"[All Fields]) OR "prevention and control"[All Fields] OR "control"[All Fields] OR "control groups"[MeSH Terms] OR ("control"[All Fields] AND "groups"[All Fields]) OR "control groups"[All Fields]) | 5,527,655 |
|  |  | 1 AND 2 AND 3 | 180 |
| ProQuest Dissertation and Thesis |  | ab(insomnia OR dyssomnia OR sleep disorders OR sleep disturbance OR sleeplessness OR sleep maintenance OR somnipathy OR sleep hygiene) | 3,300 |
|  |  | ab(transcranial electric stimulation or TES or cranial electrical stimulation OR CES OR cranial electric stimulat* OR electrotherap* OR fisher wallacestimulat* OR alpha-stim OR Neuroelectric therapy OR Transcerebral electrotherapy OR Transcranial stimulation OR tDCS OR Brain Polarization OR Electric Stimulation OR Electric Polarization OR transcranial alternative current stimulation OR tACS OR transcranial random noise stimulation OR tRNSOR transcranial magnetic stimulation OR TMS OR non-invasive brain stimulation OR NIBS) | 37,736 |
|  |  | [ab(rct or randomized control trial or randomized controlled trial)](https://search.proquest.com/recentsearches.recentsearchtabview.recentsearchesgridview.scrolledrecentsearchlist.checkdbssearchlink:rerunsearch/255F8DD530E84520PQ/None?site=pqdt&t:ac=RecentSearches) | 8,227 |
|  |  | ab(placebo or sham or control) | 505,067 |
|  |  | 1 and 2 and 3 and 4 | 71 |
| Chinese National Knowledge Infrastructure (CNKI) (Chinese database) |  | 1. **TOPIC:** (失眠 OR 睡眠障碍) | 111,816 |
|  |  | ti,ab,kw(经颅微电流刺激Or 经颅直流电刺激Or 经颅交流电刺激Or 经颅磁刺激) | 41,490 |
|  |  | ti,ab,kw(临床试验 OR RCT) | 651,137 |
|  |  | 1 & 2 & 3 | 91 |

AB = Abstract; TX = All text; ti,ab,kw = title, abstract, keywords.

**Table S1 Characteristics of TES studies**

| **Author, year, country** | **Participants** | | | | | | **TES group** | | | **Sham procedure** | **Outcome measures** | **Assessment time point** |
| --- | --- | --- | --- | --- | --- | --- | --- | --- | --- | --- | --- | --- |
|  | **Type of insomnia** | **Diagnosis** | **Age** (years, mean [SD]) | **Male%** | **Sample size** (I/C) | **Attrition rate** | **Intervention** | **Electrodes position** | **Stimulation parameters**  *Intensity, Frequency, Duration* |  |  |  |
| Cartwright & Weiss, 1975, U.S. | Primary insomnia | Sleep latency: >60 min;  insomnia ≥3 nights/week | n/a | n/a | 10 (5/5) | 0.00% | Electrosleep treatment | The electrodes were applied to the areas above the eye and the nape of the neck | I: 0.1 mA;  D: 1^st^ day: 5 mins,  2^nd^ day 10 mins,  3^rd^ - 24^th^ day: 15 mins (24 sessions) | Sham (no electrical current) | Sleep onset difficulty; Subject sleep | 2 years follow up |
| Kwon et al., 2019, South Korea | Primary insomnia | ICSD-3 | I: 62.00  [9.00]  C: 60.00 [10.00] | 25.9 | 27 (14/13) | 0.00% | CES | Ear-clip electrode | I: 0.025 mA;  F: 8 Hz;  D: 60 mins/day, 28 consecutive days (28 sessions) | Sham CES (no electrical current) | PSQI;  ISI | Mid-way (2^nd^ week), post intervention |
| Lande&Gragnani, 2013, U.S. | Primary insomnia | PSQI ≥21 | n/a | 80.7 | 57 (28/29) | 3.50% | Alpha-Stim SCS cranial electrotherapy stimulator | Ear-clip electrode | I: 0.1 mA;  D: 60 mins/day, 5 consecutive days (5 sessions) | Sham CES | Sleep log (time to sleep onset, total time slept, number of awakening) | Post intervention, 3 days and 10 days after the completion of the intervention |
| Lyon et al., 2015, U.S. | Breast cancer women with depression, anxiety, pain, fatigue, and sleep disturbances | n/a | 51.00 [0.78] | 0.0 | 167  (84/83) | 2.40% | CES | Ear-clip electrode(Alpha-Stim) | I: 0.1 mA;  F: 0.5 Hz;  D: 60 mins/day, 6-32 consecutive weeks | Sham CES (no electrical current) | GSDS | Post intervention |
| Rose et al., 2009, U.S. | Spousal caregiver for a spouse with Alzheimer’s disease | GSDS ≥10 | I: 71.94 [7.78]  C: 76.52 [5.60] | 34.2 | 39 (20/19) | 2.60% | CES | Ear-clip electrode(Alpha-Stim) | 60 mins/day,  28 consecutive days (28 sessions) | Sham CES (no electrical current) | PSQI; GSDS  Sleep diary | Post intervention |
| Rosenthal, 1972, U.S. | Patients with neurotic and personality disorders with prominent anxiety,  Depression and insomnia | Diagnosis of neurotic and personality disorders with prominent anxiety,  Depression and insomnia | I: 42.80  C: 43.50 | 9.1 | 22 (11/11) | 0.00% | Active electrosleep | anode over mastoids, cathode over orbits | I: 0.5-1.2 mA  D: 90 mins/day, 5 consecutive days (5 sessions) | Sham (no electrical current) | Sleep disturbance | Post intervention |
| Taylor Ann, et al., 2011, U.S. | People with Fibromyalgia | n/a | 50.80 [10.4] | 6.5 | 57 (19/20/18) | 19.30% | CES | Ear-clip electrode (Alpha-Stim) | I: 0.1 mA;  F: 0.5 Hz;  D: 60 mins/day, 56 consecutive days (56 sessions) | Sham (no stimulation) | GSDS | Post intervention |
| Weiss, 1973, U.S. | Primary insomnia | DSM-IV-TR; ICD-10;  Insomnia > 3 months;  insomnia ≥3 nights/week; PSQI >8 | n/a | n/a | 10 (5/5) | 0.00% | CES Electrosleep treatment | The electrodes were applied to the areas above the eye and the nape of the neck;  Above the eyes and the nape of the neck | I: 0.1 mA;  D:1^st^ day: 5 mins,  2^nd^ day 10 mins,  3^rd^ - 24^th^ day: 15 mins, 24 consecutive days (24 sessions) | Sham | EEG | Post intervention, 2 week after the completion of the intervention |
| Acler et al., 2013, Italy | Patients with post-polio syndrome | n/a | n/a | n/a | 32 (16/16) | 3.10% | tDCS | Anode: two on the scalp over the right and left pre-motor cortex (2 cm ahead C3-C4);  Cathode: Left shoulder | I: 1.5 mA  D: 15 mins/day, 15 sessionsover 3 weeks | Sham tDCS (discontinued electrical stimulation after five seconds) | PSQI | Post intervention |
| Bianchi M.S., et al., 2017, Brazil | Postmenopausal women | n/a | I: 55.30 [5.60]  C: 53.80 [5.60] | 0.0 | 30 (15/15) | 0.00% | tDCS | Anode: motor cortex M1, C3, or C4 position;  Cathode: contralateral supraorbital region | I: 2 mA  D: 20 mins/day, 10 consecutive days (10 sessions) | Sham tDCS (discontinued electrical stimulation after 30 seconds) | PSQI | 4 weeks after the completion of the intervention |
| Bimorgh et al., 2020, Iran | Patients under methadone maintenance treatment | n/a | I: 37.36 [7.63]  C: 36.00 [5.69] | 100.0 | 30 (15/15) | 10.00% | tDCS | Anode: right dorsolateral prefrontal area (F4);  Cathode: left dorsolateral prefrontal area (F3) | I: 2 mA;  D: 20 mins/session, 7 session over 2 weeks | Sham tDCS (discontinued electrical stimulation after some seconds) | PSQI | Mid-way (4^th^ session), post intervention |
| Cody et al., 2020, U.S. | Adults aged 50 and older with or without HIV | n/a | HIV-positive: 55.82 [4.34];  HIV-negative: 62.12 [10.40] | n/a | 66 (34/32) | 0.00% | tDCS+ speed of processing (SOP) | Anode: right inferior frontal cortex near F10;  Cathode: contralateral upper arm | I: 2 mA;  D: 20 mins/session, 2 sessions/week for 5 weeks (10 sessions) | Sham tDCS (discontinued electrical stimulation after 30 seconds) | PSQI | Post intervention |
| Harvey et al., 2017, Canada | Elderly with chronic pain | ISI >7 | 71.00 [7.00] | 21.4 | 16 (8/8 ) | 12.50% | tDCS | Anode: over M1, contralateral to the most painful site (C3 or C4 per the electroencephalogram 10/20 system) | I: 2 mA  D: 20 mins/day, 5 consecutive days (5 sessions) | Sham tDCS (current was applied only for the initial and final 30 seconds) | PSQI | Post intervention, 1 week after the completion of the intervention |
| Roizenblatt et al., 2007, U.S. | Patients with fibromyalgia | n/a | 53.40 [8.90] | 0.0 | 32 (11/11/10) | 0.0% | I1: tDCS of M1  I2: tDCS of left DLPFC | I1: M1 stimulation  Anode: C3  Cathode: contralateral supraorbital area  I2: left DLPFC stimulation  Anode: F3;  Cathode: contralateral supraorbital area | I: 2 mA  D: 20 mins/day, 5 consecutive days (5 sessions) | sham tDCS  (discontinued electrical stimulation after 30 seconds) | PSG | Post intervention |
| Zhou et al., 2020, China | People with major depression and insomnia | DSM-V; ICD-10; | I: 43.91 [11.20]  C: 40.45 [8.31] | 33.3 | 90 (47/43) | 0% | tDCS | Anode: LDLPFC;  Cathode: RDLPFC | I: 2 mA  D: 30 mins/day, 20 sessions over 4 weeks | sham tDCS  (discontinued electrical stimulation after 30 seconds) | PSQI; PSG | Post intervention |
| Wang et al., 2019, China | Primary insomnia | DSM-IV-TR; ICD-10;  Insomnia > 3 months;  insomnia ≥3 nights/week; PSQI >8 | I: 52.50 [10.70]  C: 55.30 [8.00] | 24.2 | 62 (31/31) | 3.20% | tACS | Forehead and both mastoid areas | I: 15 mA  F: 77.5 Hz  D: 40 mins/day, 20 sessions over 4 weeks | sham tACS (no stimulation) | PSQI | Post intervention, 4 weeks follow up |

TES: transcranial electric stimulation;CES: cranial electrotherapy stimulation;tDCS: transcranial direct current stimulation; tACS: transcranial alternative current stimulation

DSM: The Diagnostic and Statistical Manual of Mental Disorders; CCMD: Chinese Classification of Mental Disorders.

PSQI: Pittsburgh Sleep Quality Index; ISI: insomnia severity index; PSG: polysomnography; ICSD: International Classification of Sleep Disorders; GSDS: General sleep disturbance scale.

ICD: The International Statistical Classification of Diseases and Related Health Problems; DSM: The Diagnostic and Statistical Manual of Mental Disorders; ICSD: International Classification of Sleep Disorders; CCMD: Chinese Classification of Mental Disorders.

**Table S2 Characteristics of rTMS studies**

| **Author, year, country** | **Participants** | | | | |  | **rTMS intervention group** | | | **Sham procedure** | **Outcome measures** | **Assessment time point** |
| --- | --- | --- | --- | --- | --- | --- | --- | --- | --- | --- | --- | --- |
|  | **Type of insomnia** | **Diagnosis** | **Age (years, mean [SD])** | **Male%** | **Sample size (I/C)** | **Attrition rate** | **Intervention** | **Position** | **Stimulation parameters**  *Magnetic field strength (tesla),*  *(Resting) Motor threshold, Frequency,*  *Pulses/session,*  *Duration* |  |  |  |
| Anniwan, et al., 2016, China | Primary insomnia | ICD-10;  insomnia > 3 nights/week; duration > 1 months;  PSQI>7 | I: 43.56 [21.01]  C: 41.57 [22.95] | 48.3 | 60 (30/30) | 0.0% | rTMS | RDLPFC | MT: 100%;  F: 1 Hz;  P: 1500  D: 25 mins/session, 14 consecutive days (14 sessions) | Inactive coil and sound | PSQI; PSG | Post-intervention |
| Arias Pablo, et al., 2010, UK | People with Parkinson disease and insomnia | n/a | n/a | n/a | 19 (10/9) | 5.30% | rTMS | Vertex | rMT: 90%;  F: 1 Hz;  P: 100  D: 10 sessions over 2 weeks | 90° tilted coil | Actigraph, Parkinson’s Disease Sleep Scale (PDSS) | Post-intervention; 1 week after the complementation of the intervention |
| Ding, et al., 2020, China | Primary insomnia | Diagnosis and treatment of adult insomnia in China | I: 40.59 [5.14]  C: 40.67 [5.41] | 46.3 | 80 (40/40) | 0.0% | rTMS | RDLPFC | MT: 80-130%;  F: 0.5-1 Hz  D: 20 mins/session, 10 sessions over 2 weeks | 90° tilted coil and sound | PSQI; PSG | Post-intervention |
| Feng et al., 2017, China | Primary Insomnia | ICD-10;  PSQI >7 | I: 44.10 [8.30]  C: 45.10 [7.80] | 48.1 | 80 43/37) | 6.25% | rTMS | RDLPFC | T: 1.5;  rMT: 80%  F: 1 Hz;  P: 1200  D: 10 consecutive days (10 sessions) | 90° tilted coil | PSQI | Post-intervention |
| Guo, et al., 2020, China | Primary insomnia | CCMD-3 | I: 40.50 [4.30]  C: 41.50 [4.80] | 58.3 | 60 (30/30) | 0.0% | rTMS + medicine (zolpidem) | RDLPFC | MT: 80-130%  F: 0.5-1 Hz  D: 20 mins/session, 20 sessions over 4 weeks | 90° tilted coil | PSQI | Post-intervention |
| He, et al., 2009, China | Primary insomnia | DSM-IV | I1: 37.40 [16.80]  I2: 38.20 [18.60]  C: 36.60 [17.10] | 33.3 | 126 (44/42/40) | 0.0% | rTMS | At the middle of bilateral frontal/occipital/temporal cortex | T: 0.2-1.0;  F: 1 Hz  D: 30 mins/session, 10 consecutive days (10 sessions) | Invalid coil and sound | Krakow Sleep score | Post-intervention; 20 days after the complementation of the intervention |
| Hu, et al., 2014, China | People with anxiety and insomnia | CCMD-3 | 56.20 [5.70] | 47.4 | 76 (38/38) | 0.0% | rTMS + medicine (venlafaxine) | RDLPFC | T: 1.5;  MT: 80%;  F: < 1 Hz  D: 20 mins/session, 7 consecutive days (7 sessions) | Inactive coil | PSQI; PSG | Post-intervention |
| Hu et al., 2019, China | Primary insomnia | DSM-IV;  PSQI ≥ 7;  Insomnia duration ≥ 6 month;  Sleep latency ≥ 30 min;  Sleep efficiency: < 80% | I1: 71.53 [6.47]  I2: 71.10 [6.36]  C: 68.53 [6.90] | 44.4 | 90 (30/30/30) | 11.10% | rTMS | I1: RDLPFC;  I2: RN | rMT: 80%;  F: 1 Hz,  P: 1500  D: 20 sessions over 4 weeks | 90° tilted coil | PSQI; sleep diary | Post intervention |
| Huang et al., 2018, China | Patient with anxiety and insomnia | DSM-IV;  Insomnia duration ≥3 months;  PSQI ≥ 7 | I: 44.94 [11.64]  C: 45.22 [10.85] | 50.0 | 36 (18/18） | 0.00% | rTMS | right PPC (P4 electrode site)  1 | rMT: 90%;  F: 1 Hz;  P: 1500  D: 10 mins/session, 10 consecutive days (10 sessions) | Inactive coil and sound | PSQI | Post intervention, 2 weeks and one month after the completion of the intervention |
| Li mo, et al., 2016, China | Patients with dementia and sleep disorder | PSQI ≥7 | I: 78.97 [5.39]  C: 80.23 [4.89] | 41.9 | 62 (31/31) | 0.00% | Super low-frequency  rTMS + hypnotic medicine | RDLPFC | T: 1.5-6;  F: 1 mHz 20 min+  11mHz 5 min  D: 20 mins/session, 10 sessions over 2 weeks | Inactive coil | PSQI | Post intervention |
| Li, et al., 2017, China | Patients with insomnia and anxiety | CCMD-3 | 37.97 [11.54] | 45.0 | 40 (20/20) | 0.00% | rTMS + medicine (Tandospirone citrate tables) | RDLPFC | MT: 80%  D: 20 mins/session, 14 consecutive days (14 sessions) | Inactive coil | PSQI | Post-intervention |
| Li et al., 2019, China | Primary insomnia | Diagnosis and treatment of adult insomnia in China | I: 34.58 [12.13]  C: 36.28 [12.36] | 42.5 | 120 (60/60) | 0.00% | rTMS+ medicine (lorazepam) | RDLPFC | MT: 80%;  F: 0.5 Hz  D: 20 sessions over 4 weeks | 90° tilted coil | PSQI, PSG | Mid-way (1^st^, 2^nd^, 3^rd^), post intervention |
| Liang et al., 2012, China | Primary insomnia | CCMD-III;  PSQI >7 | I: 24.71 [7.52]  C: 23.89 [6.85] | 85.4 | 48 (24/24) | 0.00% | rTMS + medicine (Alprazolam) + usual care: (Alprazolam + psychological counselling + relax exercise) | Left prefrontal cortex | MT 80%;  F: 1 Hz;  P: 2400  D: 20 mins/session, 15 sessions over 3 weeks | 180° tilted coil  + with usual care | PSG | Post intervention |
| Lin Jian, et al., 2019, China | males with drug addiction and insomnia | DSM-IV | n/a | n/a | 105 (40/40/25) | 0.00% | rTMS | LDLPFC | rMT: 100%;  F: 10 Hz;  P: 2000  D: 10 mins/session, 30 sessions over 6 weeks | 90° tilted coil | PSQI | Post-intervention |
| Lin, et al., 2020 | Primary insomnia | CCMD-3;  insomnia > 3 nights/weekduration> 1 months;  Sleep latency ≥ 30 min;  PSQI≥7 | I: 52.90 [16.50]  C: 53.40 [15.40] | 44.4 | 72 (36/36) | 0.00% | rTMS | Acupoints (baihui, sishencong, benshen, shenting, anmian) | MT: 100%;  F: 1 Hz;  P: 1500  D: 25 mins/session, 28 consecutive days (28 sessions) | Inactive coil and sound | PSQI | Post-intervention |
| Liu et al., 2016a, China | Primary insomnia | CCMD-3;  PQSI >7 | n/a | n/a | 93 (31/31/31) | 0.00% | I1: rTMS  I2: rTMS+ CBT; | RDLPFC | T: 0.2-0.8;  F: 1 Hz;  P: 1800  D: 30 mins/day, 14 consecutive days (14 sessions) | Inactive coil and sound | PSQI | Post TMS intervention; 22 weeks post rTMS intervention (post CBT intervention) |
| Liu, et al., 2016b, China | Patients with alcohol abuse and insomnia | DSM-IV;  PQSI >7 | 55.30 [10.30] | 49.3 | 70 (35/35) | 0.00% | rTMS | RDLPFC | T: 2.0;  rMT: 80%;  F: 1 Hz;  P: 1600  D: 20 mins/session,28 consecutive days (28 sessions) | 180° tilted coil | PSQI; PSG | Post-intervention |
| Liu, et al., 2016c, China | Primary insomnia | DSM-IV;  PQSI >7 | 56.30[5.10] | 46.7 | 60 (30/30) | 0.00% | rTMS | RDLPFC | T: 2.0;  rMT: 80%;  F: 1 Hz;  P: 1600  D: 20 mins/session,28 consecutive days (28 sessions) | 180° tilted coil | PSQI; PSG | Post-intervention |
| Liu et al., 2018a, China | people with ketamine addiction and insomnia | ICD-10 F15;  PSQI >7 | 24-46 | 53.3 | 90 (45/45) | 0.00% | rTMS | RDLPFC | rMT: 80%-100%;  F: 1 Hz;  D: 15 mins/session, 30 consecutive days (30 sessions) | 180° tilted coil | PSQI;  PSG | Post intervention |
| Liu, et al., 2018b, China | Patients with chronic fatigue syndrome and insomnia | n/a | I: 35.40 [6.72]  C: 35.34 [6.58] | 60.0 | 70 (35/35) | 0.00% | rTMS | RDLPFC | MT: 80%;  F: 1 Hz;  P: 1800  D: 25 mins/session, 10 sessions over 2 weeks | Invalid coil and sound | PSQI | Post-intervention; 2 weeks after the complementation of the intervention |
| Meng, et al., 2017, China | Primary insomnia | ICD-10; PSQI>7 | 18-59 | 51.1 | 90 (29/30/31) | 0.00% | rTMS | right lateral PFC (RLPFC) and middle PFC | F: 1 Hz  D: 20 mins/session, 20 sessions over 4 weeks | Inactive coil | PSQI | Post-intervention |
| Shen and Wong, 2018, China | Primary insomnia | ICD-10 | I: 40.60[9.20]  C: 41. 40[8.90] | 42.9 | 98 (49/49) | 13.30% | Low-frequency rTMS | RDLPFC | MT: 80-130%;  F: 0.5-1Hz;  D: 20 mins/session, 20 sessions over 4 weeks | 20% MT | PSQI; PSG | Post-intervention |
| Xie, et al., 2020, China | Primary insomnia | Diagnosis and treatment of adult insomnia in China | I: 48.30 [6.07]  C: 48.75 [6.16] | 28.1 | 160 (80/80) | 4.40% | rTMS + medicine (zolpidem) | RDLPFC | MT: 90%;  F: 1 Hz;  P: 1400  D: 20 sessions over 4 weeks | 90° tilted coil | PSQI; PSG | Post-intervention; 4 weeks after the complementation of the intervention |
| Yan, et al., 2019, China | Primary insomnia | PSQI ≥7 | I: 32.16 [6.18]  C: 33.25 [5.35] | 47.1 | 87 (43/44) | 0.00% | rTMS + medicine (estazolam tablets) | n/a | F: <0.2 Hz  D: 30 mins/session, 10 sessions over 2 weeks | coil was placed 10-15 cm over the stimulation site | PSQI | Post-intervention |
| Yu et al., 2018, China | Primary insomnia | DSM-IV | I: 67.59 [3.05]  C: 66.07 [3.81] | 36.3 | 80 (40/40) | 6.25% | rTMS+Zolpidem | RDLPFC;  rTMS +Zolpidem | MT: 90%;  F: 1 Hz;  P: 1100  D: 20 sessions over 4 weeks | 90° tilted coil) +  Zolpidem | PSQI; PSG | Post intervention |
| Yuan et al., 2018, China | Primary insomnia | ICD-10;  Duration > 1 month;  insomnia ≥ 3nights/week | I: 52.73 [11.98]  C: 52.63 [15.18] | 43.3 | 60 (30/30) | 0.00% | rTMS | RDLPFC | MT: 90-110%;  F: 1 Hz  D: 20 mins/session, 20 sessions over 4 weeks | Sham rTMS | PSQI | Mid-way (2 weeks), post-intervention |
| Zhang et al., 2018, China | Primary insomnia | DSM-V;  insomnia ≥ 3 nights/weeks > 1 months;  PSQI: 7-15 | I: 51.30[8.70]  C: 49.80[9.20] | 14.1 | 78 (40/38) | 3.80% | rTMS treatment + Acupuncture | Left prefrontal cortex; | rMT: 100%;  F: 1 Hz;  P: 1200  D: 30 mins/session, 3 sessions/week, 12 sessions over 4 weeks | Inactive coil  +Acupuncture | PSQI; ISI; sleep diary; actigraphy diary-derived measures | Post-intervention; 2 weeks after the complementation of the intervention |

rTMS: transcranial magnetic stimulation; RDLPFC: right dorsolateral prefrontal cortex; LDLPFC: left dorsolateral prefrontal cortex;RN: raphe nuclei; PPC: posterior parietal cortex; MT: Motor threshold; rMT: resting motor threshold.

PSQI: Pittsburgh Sleep Quality Index; ISI: insomnia severity index; PSG: polysomnography;

ICD: The International Statistical Classification of Diseases and Related Health Problems; DSM: The Diagnostic and Statistical Manual of Mental Disorders; CCMD: Chinese Classification of Mental Disorders

**Table S3 Meta-analyses of sleep parameters measured by PSG and EEG of TES studies (random effect analysis)**

| **Sleep-related outcomes** | **no. of studies** | **no. of participants** | **WMD [95% CI]** | **P value** | **I^2^ (%)** |
| --- | --- | --- | --- | --- | --- |
| SE | 2 | 132 | -4.86 [17.29, 7.57] | 0.44 | 93 |
| SOL | 3 | 142 | 1.24[-10.05, 12.52] | 0.83 | 81 |
| TST | 2 | 132 | -7.75 [-42.25, 26.74] | 0.66 | 79 |

SE:Sleep efficiency; SOL: Sleep onset latency; TST: Total sleep time;

**Table S4 Univariate analyses of moderators for effects of TES on PSQI at post intervention**

| **Effect moderator** | **Coefficient** | **SE** | **Lower limit** | **Upper limit** | **z** | **p** |
| --- | --- | --- | --- | --- | --- | --- |
| **TES** |  |  |  |  |  |  |
| **PSQI (*k* = 7)** |  |  |  |  |  |  |
| Age | 0.0121 | 0.0113 | -0.0101 | 0.0343 | 1.07 | 0.2858 |
| Male% | -0.8171 | 0.5158 | -1.8280 | 0.1939 | -1.58 | 0.1132 |
| Current intensity | -0.0205 | 0.0293 | -0.0779 | 0.0369 | -0.70 | 0.4846 |
| Total number of sessions | 0.0239 | 0.0189 | -0.0132 | 0.0611 | 1.26 | 0.2066 |
| Number of weekly sessions | 0.1992 | 0.1190 | -0.0341 | 0.4324 | 1.67 | 0.0943 |
| Length of treatment/session | 0.0103 | 0.0084 | -0.0063 | 0.0268 | 1.22 | 0.2234 |

**Table S5 Meta-analyses of sleep parameters measured by PSG and actigraphy of rTMS studies (random effect analysis)**

| **Sleep-related outcomes** | **no. of studies** | **no. of participants** | **WMD [95% CI]** | **P value** | **I^2^ (%)** |
| --- | --- | --- | --- | --- | --- |
| SE | 8 | 696 | 7.91 [3.70, 12.12] | **0.0002** | **93** |
| SOL | 12 | 992 | -9.78 [-13.25, -6.31] | **<0.00001** | 90 |
| TST | 8 | 696 | 37.25 [21.51, 52.98] | **<0.00001** | 80 |
| WASO | 11 | 944 | -27.86 [-38.70, -17.02] | **<0.00001** | 96 |
| NA* | 7 | 524 | -1.06 [-1.53, -0.59] | <0.00001 | 22* |

SE:Sleep efficiency; SOL: Sleep onset latency; TST: Total sleep time; WASO: wake after sleep onset; NA: number of awakenings

*Fixed-effects model

**Table S6 Univariate analyses of moderators for effects of rTMS on insomnia at post intervention**

| **Effect moderator** | **Coefficient** | **SE** | **Lower limit** | **Upper limit** | **z** | **p** |
| --- | --- | --- | --- | --- | --- | --- |
| **SE (*k* = 8)** |  |  |  |  |  |  |
| Age | 0.0225 | 0.0278 | -0.0321 | 0.0770 | 0.81 | 0.4192 |
| Male% | 1.1171 | 2.4589 | -3.7022 | 5.9365 | 0.45 | 0.6496 |
| Number of pulses/session | -0.0000 | 0.0008 | -0.0016 | 0.0015 | -0.02 | 0.9820 |
| Total number of sessions | 0.1132 | 0.0517 | 0.0119 | 0.2145 | 2.19 | **0.0284** |
| Number of weekly sessions | -0.5695 | 0.3025 | -1.1623 | 0.0233 | -1.88 | 0.0597 |
| Length of treatment/session | -0.1254 | 0.0663 | -0.2554 | 0.0046 | -1.89 | 0.0587 |
| **SOL (*k* = 12)** |  |  |  |  |  |  |
| Age | 0.0025 | 0.0267 | -0.0499 | 0.0549 | 0.09 | 0.9250 |
| Male% | -0.9581 | 1.6844 | -4.2595 | 2.3434 | -0.57 | 0.5695 |
| Number of pulses/session | -0.0001 | 0.0008 | -0.0017 | 0.0016 | -0.08 | 0.9361 |
| Total number of session | 0.0088 | 0.0381 | -0.0659 | 0.0836 | 0.23 | 0.8168 |
| Number of weekly sessions | 0.1643 | 0.2165 | -0.2600 | 0.5887 | 0.76 | 0.4478 |
| Length of treatment/session | 0.1603 | 0.1065 | -0.0484 | 0.3689 | 1.51 | 0.1322 |
| **WASO (*k* = 11)** |  |  |  |  |  |  |
| Age | -0.0030 | 0.0399 | -0.0813 | 0.0753 | -0.08 | 0.9399 |
| Male% | -0.2776 | 3.0224 | -6.2014 | 5.6462 | -0.09 | 0.9268 |
| Number of pulses/session | 0.0004 | 0.0029 | -0.0052 | 0.0061 | 0.15 | 0.8787 |
| Total number of sessions | 0.0084 | 0.0444 | -0.0786 | 0.0954 | 019 | 0.8502 |
| Number of weekly sessions | 0.0373 | 0.2528 | -0.4581 | 0.5328 | 0.15 | 0.8826 |
| Length of treatment/session | 0.1834 | 0.1013 | -0.0151 | 0.3819 | 1.81 | 0.0702 |
| **TST (*k*= 8)** |  |  |  |  |  |  |
| Age | 0.0281 | 0.0166 | -0.0044 | 0.0606 | 1.69 | 0.0904 |
| Male% | -0.0164 | 1.3167 | -2.5971 | 2.5643 | -0.01 | 0.9901 |
| Number of pulses/session | -0.0006 | 0.0007 | -0.0020 | 0.0008 | -0.80 | 0.4247 |
| Total number of sessions | 0.0501 | 0.0543 | -0.0563 | 0.1566 | 0.92 | 0.3561 |
| Number of weekly sessions | 0.0754 | 0.2160 | -0.3481 | 0.4988 | 0.35 | 0.7272 |
| Length of treatment/session | -0.0937 | 0.0435 | -0.1790 | -0.0085 | -2.16 | **0.0311** |
|  |  |  |  |  |  |  |
| **PSQI: post treatment(*k*= 22)** |  |  |  |  |  |  |
| Age | -0.0306 | 0.0183 | -0.0664 | 0.0052 | -1.67 | 0.0943 |
| Male% | -5.1402 | 2.2335 | -9.5179 | -0.7626 | -2.30 | **0.0214** |
| Number of pulses/session | -0.0028 | 0.0012 | -0.0051 | -0.0005 | -2.41 | **0.0161** |
| Total number of sessions | -0.0976 | 0.0330 | -0.1622 | -0.0330 | -2.96 | **0.0031** |
| Number of weekly sessions | -0.0142 | 0.2176 | -0.4406 | 0.4123 | -0.07 | 0.9480 |
| Length of treatment/session | 0.1138 | 0.0402 | 0.0351 | 0.1925 | 2.83 | **0.0046** |
| Stimulation site (Reference : DLPFC) |  |  |  |  |  |  |
| Other sites | -0.4245 | 0.6187 | -1.6371 | 0.7882 | -0.69 | 0.4927 |

SOL: sleep onset latency; TST: total sleep time; SE: sleep efficiency; WASO: wake after sleep onset;

k: number of studies

**Table S7 Adverse events summary**

| **Study name** | **Intervention** | **Sample size** | **any AE** | **dizziness** | **headache** | **pain** | **fatigue** | **itchiness** | **discomfort** | **muscle spasm** | **constipation** |
| --- | --- | --- | --- | --- | --- | --- | --- | --- | --- | --- | --- |
| Acler et al., 2013 | tDCS | 16 | 1 | 1 | - | - | - | - | - | - | - |
|  | sham tDCS | 16 | 0 | - | - | - | - | - | - | - | - |
| Bianchi M.S., et al., 2017 | tDCS | 15 | 2 | - | - | - | - | 2 | 2 | - | - |
|  | sham tDCS | 15 | 0 | - | - | - | - | - | - | - | - |
| Zhou et al., 2020. China | tDCS | 47 | a few | - | - | a few | - | - | - | - | - |
|  | sham tDCS | 43 | 0 | - | - | - | - | - | - | - | - |
| Huang et al., 2018 | rTMS | 18 | ? |  | 5 | 6 |  |  |  |  |  |
|  | sham rTMS | 18 | ? |  | 3 | 4 |  |  |  |  |  |
| Yu et al., 2018 | rTMS | 37 | 3 | - | - | 3 | - | - | - | - | - |
|  | sham rTMS | 38 | 0 | - | - | - | - | - | - | - | - |
| Yuan et al., 2018 | rTMS | 30 | 5 | - | - | 1 | - | - | 3 | 1 | - |
|  | sham rTMS | 30 | 0 | - | - | - | - | - | - | - | - |
| Lin Jian, et al., 2019 | rTMS | 40 | 3 | 3 | - | 3 | - | - | - | - | - |
|  | sham rTMS | 40 | 1 | 1 | - | 1 | - | - | - | - | - |
| Yan, et al., 2019, China | rTMS | 20 | 2 | 1 | - | - | - | - | - | - | 1 |
|  | sham rTMS | 20 | 9 | 2 | 5 | - | - | - | - | - | 2 |
| Xie, et al., 2020, China | rTMS | 77 | 12 | 7 | 3 | 2 | - | - | - | - | - |
|  | sham rTMS | 76 | 9 | 6 | 2 | - | 1 | - | - | - | - |
| Guo, et al., 2020, China | rTMS | 30 | 2 | - | - | 1 | - | - | 1 | - | - |
|  | sham rTMS | 30 | 0 | - | - | - | - | - | - | - | - |

**Table S8 Meta-analysis of adverse events of NIBS**

| **Events** | **No. of studies** | **Event/total**  **(Intervention)** | **Event/total**  **(sham group)** | **Combined effect** | | **I^2^(%)** |
| --- | --- | --- | --- | --- | --- | --- |
|  |  |  |  | RR (95% CI) | P value |  |
| Any adverse event | 8 | 3/265 | 19/265 | 1.51 [0.90, 2.53] | 0.12 | 42 |
| Dizziness | 4 | 12/153 | 9/152 | 1.31 [0.58, 2.94] | 0.52 | 0 |
| Headache* | 3 | 8/115 | 10/114 | 0.81 [0.34, 1.89] | 0.62 | 50 |
| Pain* | 6 | 16/232 | 5/232 | 2.58 [1.14, 5.84] | 0.02 | 0 |
| Discomfort | 3 | 6/75 | 0/75 | 5.00 [0.89, 27.97] | 0.07 | 0 |

NIBS: Non-invasive brain stimulation

*The adverse events reported in the transcranial magnetic stimulation studies (rTMS) were included in the meta-analysis (fixed-effect analysis).

**Figure S1 Risk of bias summary**

**
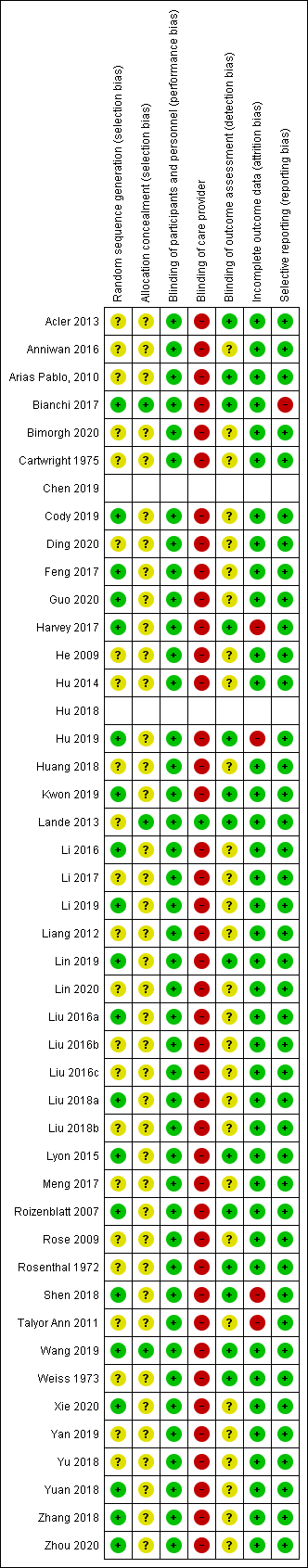
**

**Figure S2Risk of bias summary graph**

**
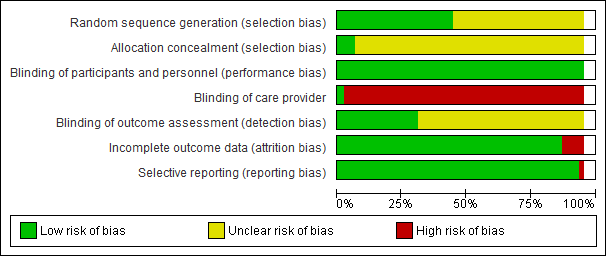
**

**Figure S3 Sensitivity analysis examining the effect of TES on people with primary insomnia at the post intervention (PSQI)**

**
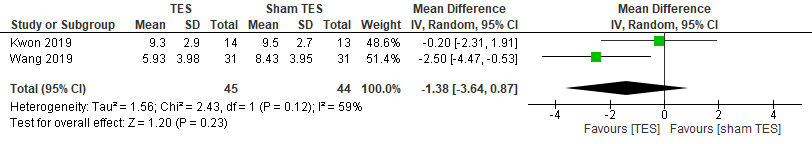
**

**Figure S4 Sensitivity analysis examining the effect of rTMS on people with primary insomnia at the post intervention (PSQI)**


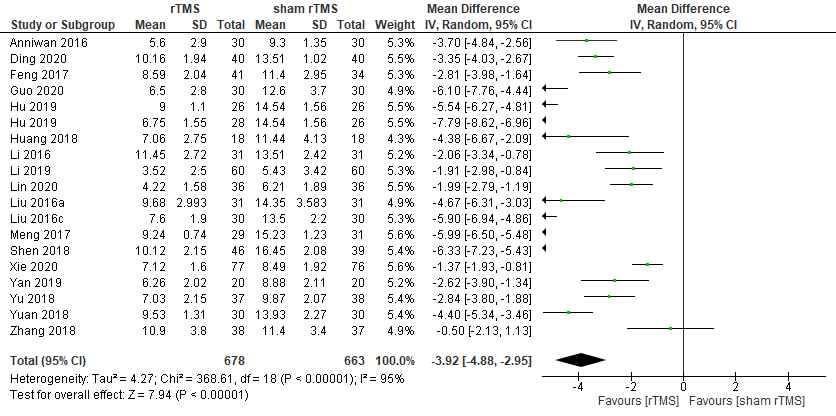


**Figure S5Sensitivity analysis examining the effect of TES on people with primary insomnia at the post intervention(PSQI) (Excluding one study with a high risk of bias)**


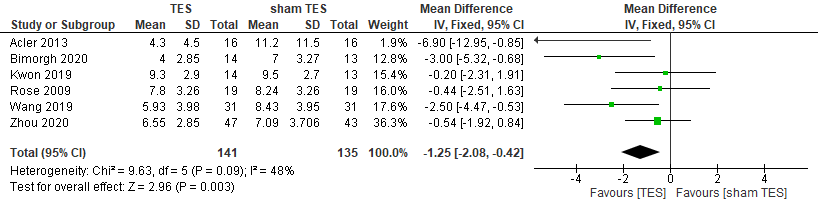


**Figure S6Sensitivity analysis examining the effect of rTMS on people with primary insomniaat the post intervention(PSQI) (Excluding two studies with a high risk of bias)**

**
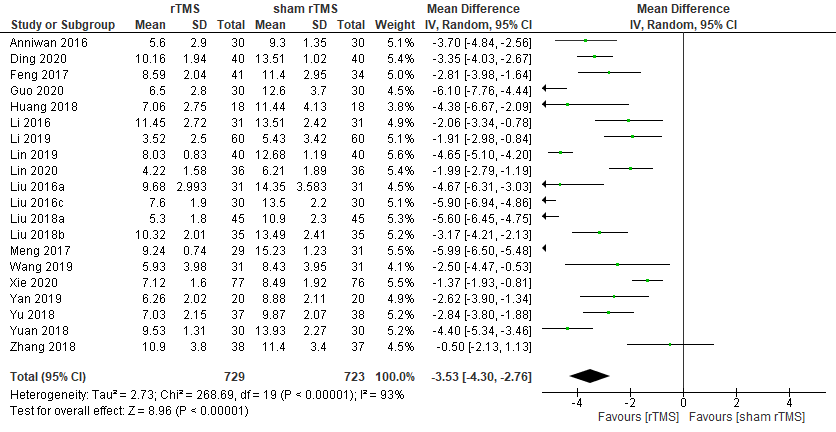
**

**Figure S7 Funnel Plots estimation of publication bias in meta-analysis of the effect of rTMS on the WASO to the sham group at the post intervention**

**Figure S8 Funnel Plots estimation of publication bias in meta-analysis of the effect of rTMS on the SOL to the sham group at the post intervention**

**Figure S9 Funnel Plots estimation of publication bias in meta-analysis of the effect of rTMS on the PSQI score to the sham group at the post intervention**
